# The I-ACTED study (investigating action civics training through an experimental design): a cluster randomized controlled trial of a school-based action civics education intervention on adolescent wellbeing

**DOI:** 10.1186/s12889-025-24838-y

**Published:** 2025-10-16

**Authors:** Alison K. Cohen, Jason C. Fitzgerald, Grisel Trejo, Isabella U. Yalif, Paul D. Wesson, Mark Wolfson, Parissa J. Ballard

**Affiliations:** 1 Department of Epidemiology & Biostatistics, University of California San Francisco, San Francisco, CA USA; 2 Department of Curriculum & Instruction, Monmouth University, New Jersy, USA; 3 Department of Family & Community Medicine, Wake Forest University School of Medicine, Winston-Salem, NC USA; 4 Vanderbilt University, Nashville, TN USA; 5 Department of Social Medicine, Population, & Public Health, University of California Riverside, Riverside, CA USA

**Keywords:** Action civics education, Adolescents, Randomized controlled trial, Schools, Wellbeing

## Abstract

**Background:**

Observational studies have found that youth civic engagement is associated with positive mental health, education, and socioeconomic outcomes. However, access to civic opportunities is not evenly distributed. Many classrooms in the United States of America (USA) do not have access to high-quality civics education. Action civics approaches to civic education prepare students for civic engagement by developing the necessary civic skills, knowledge, and character. Through action civics, classes take action on a real-world issue students choose together. Some evidence suggests that action civics may positively affect participants’ wellbeing through the feelings of civic connection and empowerment. The aim of this study is to investigate, through a randomized controlled trial, the impact of a school-based action civics education intervention on civic and wellbeing outcomes, and the mechanisms of any impact observed, among middle and high school students in the USA.

**Methods:**

This study uses a cluster-randomized trial with a waitlist-control design. We are randomizing at the school level, implementing the intervention at the teacher/classroom level, and measuring outcomes at the student level. We are recruiting social studies, civics, government, and related subject teachers, across both middle and high schools, from across the USA, leveraging network ties and referrals to invite teachers/schools to participate. We aim to recruit a sample of around 1,500 students. Intervention group teachers will receive action civics curricular resources they can incorporate into their regular teaching, while the control group will not receive the curricular resources until 12 months later and will continue with their teaching as planned. Students will fill out surveys at the beginning and end of the semester, and will be invited to complete a survey six months later. Surveys will assess civic, wellbeing, demographic, and other related variables.

**Discussion:**

This study is one of the first randomized controlled trials to assess the impacts of action civics curricular materials on civic and wellbeing outcomes. The study will strengthen our understanding of the impacts of action civics education, with implications for the quality and adoption of civic curricula nationwide.

**Trial registration:**

NCT04514133 (date of registration September 25, 2020).

**Supplementary Information:**

The online version contains supplementary material available at 10.1186/s12889-025-24838-y.

## Background

Civic engagement may benefit wellbeing [1–5]. Civic engagement is multidimensional and includes behavior, knowledge, emotion, and skills in the civic domain [6]. For youth (in this study considered as young people between the ages of 10–18), engagement in civic activities can take many forms, including volunteering, activism, and electoral forms of engagement [7] and can take place in various settings such as in and outside of school [8]. Studies have found that some civic activities such as volunteering are positively associated with mental health[1, 2, 5, 9], positive youth development outcomes such as personal and social identity development[3, 9], and wellbeing [3, 4, 9]. The direction of associations between other forms of civic activities, such as activism, and mental health and wellbeing [2, 10] are still unclear [1, 5, 9, 11–13]. Additionally, the evidence is less clear about associations between different forms of civic engagement and physical health [2, 5]. 

While engagement in civic activities may be beneficial for young people, and is a core right and responsibility of participating in a democratic society, access to opportunities to support youth civic engagement are inequitably distributed [14]. For example, high-quality civics education is not equally available to all students in the USA [15]. Racial/ethnic disparities in civic education are evident, whereby students of color have fewer opportunities to engage civically at their high school, and students from families with lower SEP also have fewer civic engagement opportunities in school [16, 17]. 

School-based interventions are an efficient and effective way to reach adolescents [18–21]. With US public school students spending on average 6.64 h a day at school[22], the longest amount of time they spend on an any activity, besides sleeping[23], and public schooling being universally accessible, schools are a context for reaching most USA youth. Given this, school-based interventions have high reach and can have greater efficacy than interventions in other settings (e.g., home-based interventions) [19]. 

Action civics education—the school-based intervention we employ in this study—is an applied approach to civic education that is student-centered and project-based [24–26]. It aims to develop students’ civic skills, knowledge, and character through active engagements in civic processes. In action civics, students work as a class to take collective real-world action about an issue they are interested in[25, 27]. According to a recent systematic review[26], experiential civics education approaches like action civics have been a growing area for study. Existing studies on action civics curricula have shown that following an action civics program, students felt more informed on civics, gained trust in political institutions, and increased their civic participation and engagement [28–30]. Action civics may increase civic participation and sense of community, which may build social connection, contribution, empowerment, and civic readiness, which could potentially in turn improve wellbeing [31]. Observational studies exploring health outcomes directly following action civics education found that participants experienced increased physical health, and reported no changes in mental health [31, 32]. Taken together, evidence suggests that participating in action civics might increase participation in civic activities and improve wellbeing. However, to the best of our knowledge, the present study is one of the first randomized controlled trials to test this hypothesis.

This paper reports on the protocol for our study attempting to answer the question: does a school-based action civics education intervention affect civic and wellbeing outcomes among adolescents (ages ~ 10–18) attending middle and high schools in the USA? In our randomized controlled trial (RCT), participating schools (those which have at least one participating teacher) are randomized for teachers to receive professional development resources regarding action civics education that semester (intervention group) or one year later (waitlist control group). Students in the participating teachers’ classes are then invited to enroll in the study and complete surveys at the beginning and end of the semester in which their school is participating in the study. We hypothesize that students who are in intervention group schools will report more positive civic and wellbeing outcomes than students who are in control group schools.

## Methods

### Trial design

This study is using a cluster-randomized trial with a waitlist-control design. Randomization occurs at the school level, intervention implementation occurs at the teacher level, and outcomes measurement at the student level; students are clustered within schools. Our intention with randomization at the school level as opposed to the teacher level is to reduce the risk of spillover effects; if two teachers at the same school had different treatment assignments, it is plausible that they might be in communication with each other, whereas two teachers at different schools are meaningfully less likely to be in communication with each other about their study participation.

The intervention offers a teachers online instruction on the components and ideas behind action civics as well as an online hub where they can find, edit, and share action civics instructional materials. All online instruction and instructional materials were designed for middle and high school classrooms. Teachers can then choose to implement changes to their curriculum accordingly. Control teachers are free to do whatever they would have otherwise done. This study design uses the superiority framework.

### Participants and eligibility criteria

The I-ACTED study aims to recruit a sample of social studies, civics, history, government, and related topics teachers, both at the middle and high school level, from across the USA. School inclusion criteria are that there is at least one interested teacher who: (1) currently teaches middle or high school students, (2) teaches social studies, civics, history, government, and related topics, (3) agrees to be randomly selected into the intervention or control group, and (4) agrees to invite students in the participating classroom(s) to complete surveys. This study is also recruiting those students from participating teachers. Student inclusion criteria are that students must be (1) at least 10 years old, (2) a middle or high school student, and (3) currently enrolled in a course with a participating teacher from a school participating in the study. No concomitant interventions are prohibited during this trial.

### Recruitment process and informed consent

Recruitment for this study takes place in two ways. First, we use a network-based approach to invite districts, schools, and teachers to the program. Study staff build ties to networks of social studies administrators, district officials, teachers, and principals, and then share the study information accordingly. This includes connecting with appropriate distribution lists from school districts or graduate programs, for example, to distribute study information. Second, we use a referral process where current participants and others in the education community can receive a $100 gift card to disseminate the study information to at least 8 teachers who are eligible for the study and/or district social studies leads and/or school administrators.

As part of the recruitment process, we share study information sheets and flyers that briefly describe the study when reaching out to administrators or teachers. Study staff are also available to answer questions of prospective teacher participants, and/or their administrators. Once interest is expressed, the project coordinator contacts the interested teacher for information about their eligibility. If eligible, study staff attain administration approval by contacting the appropriate administrator (identified by the teacher) such as Social Studies Supervisor, district offices, and/or superintendents. In some instances, depending on district policies and preferences, the administrator grants approval before teachers are invited to participate. After teachers enroll in the study, we invite their students to participate. We work closely with participating schools and teachers to ensure successful student recruitment procedures (e.g., materials describing the study, distribution of assent/consent document as applicable).

Given the onset of the COVID-19 pandemic, our expected recruitment period and expected recruitment rates have shifted. We extended the planned recruitment period accordingly to account for lower recruitment rates as teachers and schools encountered other substantial challenges in their work. Our current planned recruitment period is the 2021–2022 school year through the 2024–2025 school year. We monitor recruitment during the trial in terms of schools enrolled each semester, as well as schools that complete interest forms but do not convert to enrolling in the study.

This study presents no more than minimal risk to students and teachers and it is not practicable to obtain signed informed consent/assent. Therefore, we obtained a waiver of signed informed consent/assent for participation for both the teachers and students from our IRB, with the exception of districts that have policies mandating active parental consent. Teacher participants sign a Memorandum of Understanding with information about the study. Student participants receive an information letter that includes all details about the study at a reading level appropriate for their age. The letter describes the general purpose of the study, requirements of participation, length of participation, and risks. The information letter also delineates that participation is purely voluntary, and that they may decline to participate, or withdraw from the study at any time, and that study participation will not result in any negative outcomes. Furthermore, the student participants have the option to indicate “yes” or “no” to agree to participate. Parents/legal guardians receive an information letter and opt-out form that explains the study and allows them to opt out of the study on behalf of their children either on paper or electronically. In districts requiring active parental consent, we provide a parental/guardian consent letter and require parental/guardian permission in order for their students to participate. We send parental/guardian opt-out letters home with the students. Parents/guardians have the option to fill out the paper form or complete an online RedCap form. Study staff manage and track these responses. To reduce data collection burden, we do not request parents/guardians provide the reason for opting out. All forms have study staff contact information for questions. The student information letters and paper opt out forms are available in Spanish, if requested by the school, teacher, and/or parent/guardian. The online parent/guardian opt out form asks for language preference.

Given a low likelihood of adverse effects, we were approved by our Institutional Review Board to conduct our study without a data monitoring board and without a planned compensation process for anyone who experiences harm from study participation.

### Sample size

To estimate appropriate sample sizes for this study, we conducted a power analysis in Stata using the clustersampsi estimator. Previous studies of related outcomes from participating in an action civics intervention suggest that it is reasonable to expect an effect size of 0.15–0.2 or greater [29, 31]. If we have an average cluster size of 40 students (assumes all schools enrolled have two classes of students) and 50 schools, we will be 80% powered to detect an effect size of 0.15. If we have an average cluster size of 20 students (the most conservative estimate) and 50 schools, we will be 81% powered to detect an effect size of 0.2. Therefore, we believe this design will give us reasonable power to detect effects. Additionally, we anticipate that the intra-class correlation coefficient will be no higher than 0.01 in our sample. Previous research points to low intra-class correlation coefficients [28]. Based on the approximations above, we assume an average class size of 30 students across 50 classrooms in roughly 45–50 schools, leading to a sample of around 1,500 students. We will have two arms of the study (treatment and control) and expect to have 25 schools (clusters) per arm of the study. We anticipate that the number of students in each cluster (school) will range from 20 to 50.

Data from initial recruitment efforts suggest that there are typically multiple classrooms per school that participate, leading to approximately 70 students per school. For our updated power calculations, we assume 13 schools (clusters) per study arm, with 70 students per school, and again an intraclass correlation of 0.01. Given these calculations, we are 88% powered to detect an effect size of 0.2 in this study.

### Randomization

This study uses a cluster-randomized trial with a waitlist-control design. We are using block randomization with a 1:1 allocation ratio. Each semester, we group the most similar schools together in a block (e.g., same age range, similar geographic region), keeping blocks as small as possible (e.g., 2–3 schools). Within each block, schools are then randomized to participate in the intervention or the control group; specifically, we use a random number generator in Microsoft Excel and the higher number is assigned the control and the lower number is assigned the intervention. Randomization procedures and assignments are conducted at Wake Forest University School of Medicine by a designated study staff person; only that staff person and the person helping deliver the intervention to teachers in the intervention group know the assignment status for each school; the assignment status is otherwise inaccessible to the PIs. Study staff communicate assignments to teacher participants and intervention group teachers are given action civics curricular materials.

### Procedure and data collection methods

**Fig. 1:**
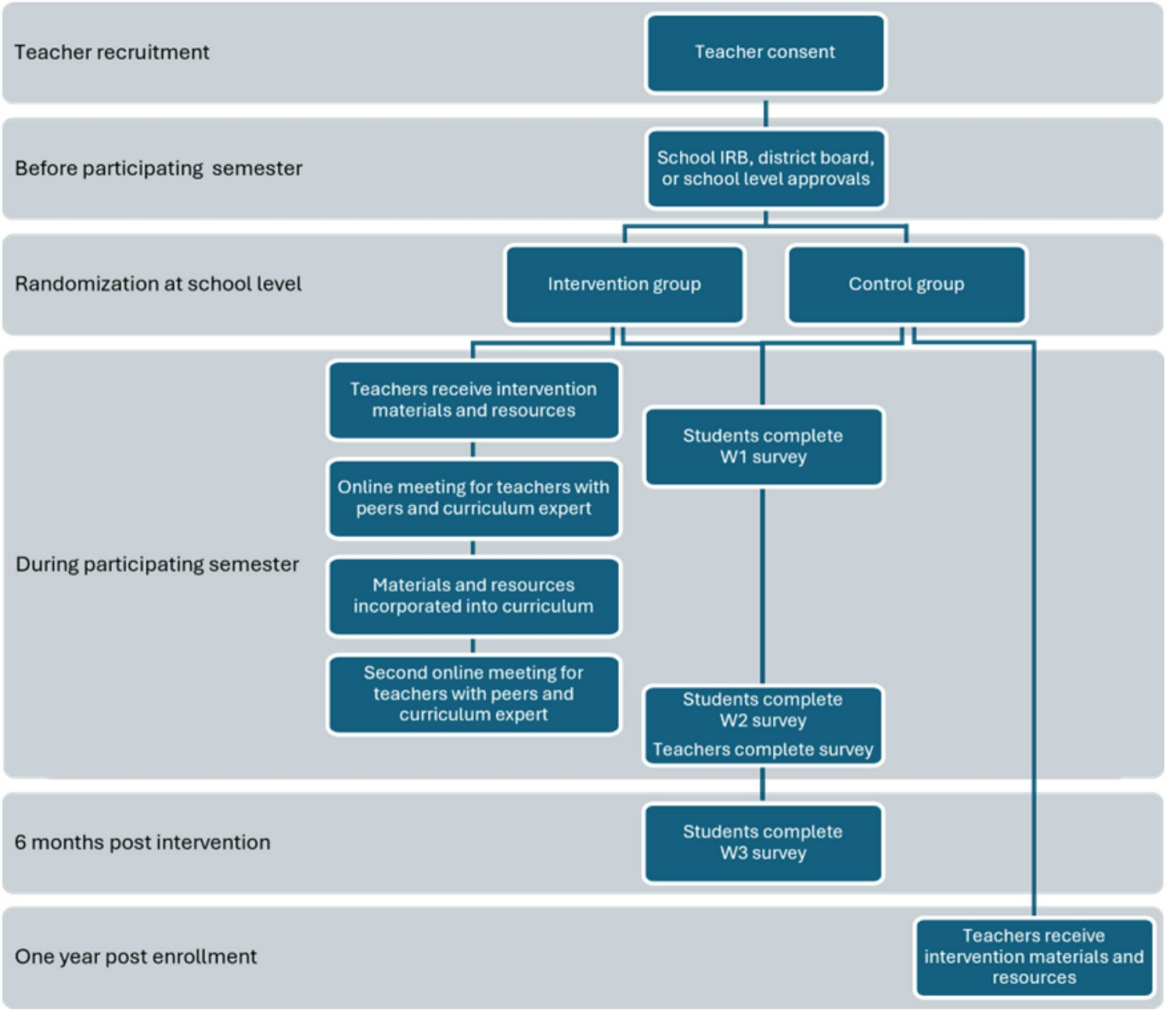
Study procedure

After teachers agree to participate in the study, study staff review the study procedures and expectations with them. This includes information about data collection and, for those randomly assigned to the intervention group, curriculum resources and training materials. Teachers inform students enrolled in their courses (both treatment and control) about the study at the beginning of the semester and, depending on district requirements, they distribute parent information letters and either opt-out forms or active parental consent forms. For districts that do not require active parental permission, students without a signed parental opt-out form can assent to participate. For districts that require active parental permission, a signed parental consent is required before students are allowed to assent to the surveys. Teachers are asked to set aside one class period near the beginning of the semester (“pre” survey Wave 1) and one class period near the end of the semester (“post” survey Wave 2) to allow students to complete the surveys (online or on paper); if district policies do not permit this, participants can also complete the surveys outside of school. Teachers in the intervention group receive action civics training and are encouraged to use the provided curricular resources throughout the semester in their teaching (Fig. 1).

We collect contact information during wave 1 and 2 from the students to facilitate an exploratory, wave 3 survey follow up. This includes personal and school email, phone number and/or address. Students will be invited by study staff to participate in a third follow-up survey six months after the 2nd survey using the contact information provided via text, call, and email. Wave 3 surveys will take place outside of the classroom setting and will be administered either online (via RedCap) or through paper surveys (via mail) by study staff. Students whose parents have “opted-out” or have not signed an active parental consent (depending on school district) will not be contacted by project staff.

Any adverse events that occur during our study will be reported to our Institutional Review Board. Given the low risk nature of the study, there is not a formal audit process.

All surveys are administered using RedCap. REDCap is an online application used to create and administer online surveys for data collection. Study staff manage and track these responses. The RedCap software assigns a unique Participant ID number to each student. The study team tracks the number of non-participants.

Participating teachers in both groups will receive up to $350 in gift cards as an incentive for their participation ($150 upon distribution of the first survey to their class (and, for intervention teachers, accessing the intervention materials) and $200 upon distributing the second survey to their class and completing a short survey about their semester). The short survey teachers complete at the end of their semester of participation provides data on what they taught that semester, to inform potential secondary analyses regarding the effect of the treatment on the treated and to assess any potential spillover.

Each semester, students who provided contact information and completed at least half of the survey will be entered into a drawing to receive a $50 gift card.

### Blinding

We are taking a triple-blind approach. The participants (students) do not know which study group they are in, and the people carrying out the statistical analysis do not know what study group participants are in. We are having as few members of the study team as possible know the treatment assignment– specifically, only the research staff person making the randomization assignments and communicating the assignment to the teacher participants, and the individuals involved helping teachers assigned to the intervention group implement the intervention. Because the study is co-led, we designated one co-PI who can know the treatment assignments if needed (e.g., if any emergency were to occur in the study, if all other staff who know the treatment assignment are permanently incapacitated simultaneously); however, this has not yet been utilized. We plan to write up the findings, based on a pre-registered analysis plan, before unblinding our results.

### Data management

Data are collected and managed using REDCap to create a secure database. All surveys completed online, which include personal information like names and contact information, are automatically part of this REDCap database. We provide the option of paper surveys to any teachers who prefer paper to online surveys; any teachers who opt for this approach would be asked to store any paper surveys in a locked file box or cabinet until they are mailed or picked up by study staff.

REDCap’s data security measures are extensive, including password protected log-ins for study staff who access the database. During data management and cleaning, all participants’ names will be replaced with their assigned IDs to produce a completely anonymous dataset. Following data collection, subject identifying information will be destroyed three years after closure of the study. Data access will be limited to study staff who have received IRB approval to access identifiable data. Data and records will be kept locked and secured, with any computer data password protected. No reference to any individual participant will appear in reports, presentations, or publications that may arise from the study.

Data and statistical code will be available based on reasonable request.

### Intervention

#### Intervention group

Teachers are provided with free Action Civics curriculum on a nonpartisan civics education framework[24], which includes three components: (1) a self-paced asynchronous action civics online training, (2) access to in-classroom resources, and (3) an online community of teachers connected to the study. The action civics online training comes in two forms. The first, known as Kick Start Action Civics, was provided by Generation Citizen and enabled teachers to learn both the conceptual underpinnings of action civics programming and the core steps for implementing action civics in the classroom setting. After this training material was taken offline, the second online action civics training materials were developed that share similar content. These include videos on engaging students in understanding local, state, and national issues and a digital civics toolkit which provides instruction that mirrors the Kick Start action civics process; these videos were provided with the same intention of providing a self-paces asynchronous action civics online training for teachers. The in-classroom resources were delivered via a Google Classroom site, which housed supporting materials related to Driving Questions, Student Learning Outcomes, ISTE [33] and C3 (College, Career, and Civic Life) standards[34], and a host of outlines and graphic organizers for instructional use. Teachers are provided access to these materials but are not obligated to use any of them and are empowered to edit and modify resources to fit their classroom needs; given this, there are no additional criteria for modifying or discontinuing the intervention. Twice per semester, teachers in the intervention groups are invited to an online meeting, where they can share resources, ask for help/support, and learn what other classrooms were doing in through their action civics programming.

All intervention materials are provided to teachers at no cost. Throughout the semester, study staff are in regular communication with teachers to ensure their understanding of what study participation entails and to provide support.

#### Control group

Teachers in the control group do not receive training and curricular resources and continue with their teaching as planned. One year after their participation in the study, teachers in the control group receive the action civics curricular resources, which they are then free to use.

### Outcome measures

All outcome measures will be self-reported by student participants, via survey. We are using an intent-to-treat design, so we will collect data from all participants, regardless of whether or not their teacher implemented the intervention (for those randomized to the intervention group). In general, we chose outcomes that could plausibly be affected by participation in action civics education, and that were validated in our participants’ age range and context whenever possible. Our planned analyses focus on changes in outcomes from wave 1 to wave 2 among intervention group participants, as compared to changes among control group participants.

#### Primary

Civic outcomes:

(A) Civic engagement – measured using an adapted version of the Youth Inventory of Involvement [35].
(B) Sense of community – measured using the Community Connection Scale from the California Healthy Kids Survey [36].

Wellbeing outcomes:

(A) Physical and mental health – measured through self-reported items from the National Longitudinal Study of Adolescent Health (Add Health) [37] and the CES-D[38].
(B) Behavioral health – measured using adapted items from the TAPS-tool [39] to assess substance abuse.
(C) Social health – measured through school absences [40] and purpose [41].
(D) General wellbeing – measured through the WHO5 Wellbeing index [42].

### Moderators

(A) Adolescents’ background characteristics – assessed using measures from Add Health [37].
(B) Experiences in action civics – measured through an adapted version of the Inventory of Service Experiences [43].
(C) Experiences in society – measured through perceived marginalization [44] and attention to political news [45].

### Mediating variables

The following are the mechanisms assessed as potential explanations of the links between civic engagement and sense of community with health and wellbeing.

(A) Psychological empowerment – measured through psychological empowerment[46], civic self-efficacy[29], and the abbreviated Sociopolitical Control Scale [47].
(B) Sense of meaningful contribution — measured with an adapted version of the contribution to organization scale [48].
(C) Social connectedness — measured with the social connectedness to peers and school subscales [49] and sense of belonging scale [50].
(D) Civic readiness and skills (captures adolescents’ ability to mobilize to bring about change) – measured as sociopolitical control, leadership skills[47], and civic and political knowledge [29].

### Data analysis

We plan to conduct an intent-to-treat analysis, which means that all randomized participants, regardless of protocol adherence, will be included in the main analyses. For our main analyses, we will use multi-level random effects multivariable regression models with maximum likelihood estimation to test the impact of the action civics intervention participation on our primary civic and wellbeing outcomes at the post time point (wave 2), controlling for civic and wellbeing outcomes at wave 1. A third multi-level multivariable regression will use the wellbeing focused regression model developed for the analysis listed directly before this, and add interaction terms for the moderator variables specified above to test for effect modification of adolescents background characteristics, experiences in action civics, and experiences in society on the association between the intervention and wellbeing outcomes.

In our planned multivariable regression models, we will account for clustering using a three-level random effects model (to account for clustering of multiple time points within students, and students nested within schools). The randomization design is designed to balance both measured and unmeasured confounding, so that the intervention and control groups are exchangeable. However, this does not always occur in practice; to test this, we plan to examine the distribution of covariates at baseline (e.g., gender, race and ethnicity, grades in school, semester of enrollment in study) to see if they are evenly divided between intervention and control. If any imbalances are present, we plan to include those covariates in our multivariable regression models.

We will test mediation using a Structural Equation Modeling (SEM) approach to assess the different potential pathways from civic engagement and sense of community to health and wellbeing via the mediating variables. In addition, we will model constructs as latent variables to combine measures of the same constructs meaningfully.

We anticipate having missing data, including, for example, if a student is absent on the day that their class completes their survey. It may also be that a student opts to not respond to certain items within our survey. We plan to use multiple imputation approaches to handle missing data in our analyses.

Per our Institutional Review Board and funder reviews, due to the low risk of this trial, it was determined a data monitoring committee was not needed. Given the low risk, it was also determined to not be necessary to have interim analysis and stopping guidelines.

## Discussion

This study was originally planned before the COVID-19 pandemic began for a fall 2020 start to recruitment, which was postponed due to the impacts of the COVID-19 pandemic on teaching and learning in schools in the USA. Our recruitment instead began recruiting for fall 2021 participation. Multiple facets of the COVID-19 pandemic, including COVID-19 surges, increased teacher burnout, and limited teacher, school, and district resources for research participation coupled with increasing regulations on school-based research have affected both recruitment and data collection activities to date.

We plan to disseminate findings regardless of the magnitude or direction of effect. Our dissemination plans include sharing the results with participating teachers and schools, as well as for any student participants for whom we have current contact information. We also plan to share findings to academic audiences (e.g., via publications in peer-reviewed academic journals, conference presentations) and other interested audiences. This articles and presentations will be authored by members of the research team only and will not include professional writers. Any dissemination activities need only to be approved by the researchers involved; there are no other approval processes for dissemination.

## Supplementary Information

Supplementary Material 1

## Data Availability

No datasets were generated or analysed during the current study.

## Abbreviations

I-ACTED: Investigating Action Civics Training through an Experimental Design
RCT: Randomized controlled trial
SEP: Socioeconomic position
USA: United States of America
